# Building sustainable capacity for better access to diabetes care in low‐resource settings: A critical review of global efforts and integrated strategies

**DOI:** 10.1002/hcs2.89

**Published:** 2024-03-25

**Authors:** Emmanuel Lamptey

**Affiliations:** ^1^ Department of Nursing, Faculty of Health and Allied Sciences KAAF University College Buduburam Ghana

**Keywords:** sustainable, capacity, access, diabetes, low‐resource settings, critical

## Abstract

The alarming state of global insulin access in low‐resource settings presents a major barrier to diabetes care. A comprehensive review of these challenges is lacking at the global level. To address this weakness, enhance affordability and build capacity for a more sustainable approach to scaling up access. This review analyzes the specific issue of inconsistent access to insulin in low‐ and middle‐income countries. Using this analysis, we mapped the scope and intensity of issues such as the unaffordability and unavailability of insulin. We also identified six innovative and integrative strategies for increasing and securing accessibility in the areas of policy making, marketing, clinical practice, health education, domestication, and multisectoral approaches.

## Graphical abstract

The alarming state of global insulin access in low resource settings presents a major barrier to diabetes care for patients living with diabetes. A comprehensive review of these challenges is lacking at the global level. To address the weakness, enhance affordability and build capacity for a more sustainable approaches to scaling up access. This review analyzes the specific issue of inconsistent access to insulin in low and middle‐income countries. By this analysis, we mapped the scope and intensity of the issue and identified innovative and integrative strategies to increasing and securing accessibility.

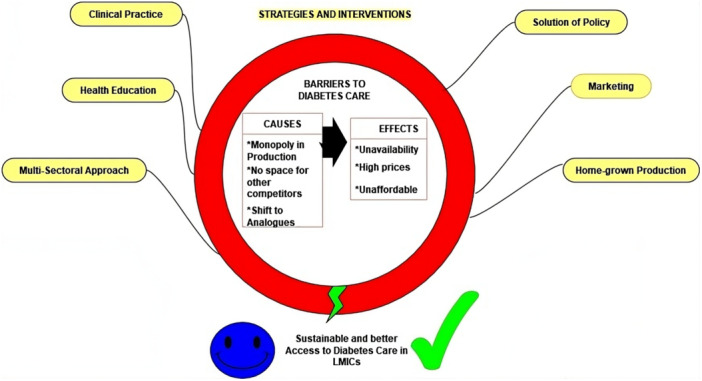

**Abbreviation**
WHO: world health organization

## 1 INTRODUCTION

Diabetes, a chronic metabolic disease characterized by high levels of blood sugar, is an urgent global health problem and is ranked as the third leading risk factor for morbidity and mortality [1, 2]. The most common type (type 2 diabetes) occurs when the body becomes resistant to insulin, unlike type 1 diabetes, in which the pancreas produces no or little insulin by itself [1]. Whether type 1 or type 2 diabetes, there is too much glucose in the blood, and when this is not controlled, it leads to serious damage to many of the body's systems, especially the nerves, blood vessels, heart, and kidney [3]. When poorly controlled, it leads to the development of chronic complications, such as blindness, kidney failure, heart attack, stroke, and lower limb amputations, with enormous healthcare costs [1]. The economic burden of diabetes and its related complications was $327 billion in the United States in 2017 and $966 billion globally in 2021 [4]. The number of people living with diabetes rose from 108 million in 1980 to 422 million in 2014, and the increase in number was seen more in low‐ and middle‐income countries than in high‐income countries [1].

Diabetes was the direct cause of death in approximately 1.5 million people in 2019, and most of these deaths occurred before the age of 70 years [1]. Between 2000 and 2019, a 3% increase in age standardized mortality has been recorded [1]. According to current statistics from the IDF Atlas, the number of people living with diabetes in 2021 is approximately 537 million, and this figure is projected to rise to 643 million by 2030 [4]. People with diabetes require ongoing access to care, education, and essential medications to manage their condition and its complications. Many of them depend on insulin and other medications, especially those with type I diabetes [1]. Those with type 2 diabetes are equally affected because many depend on insulin and other medications to manage the condition [1]. Controlling the illness reduces health risks to all individuals and provides significant global benefits because the condition affects every single body organ [5, 6]. Global efforts ensure that social support and diabetes‐related care are in correlation or capable of predicting and promoting healthy behaviors [7]. The disease needs to be paid more attention than before due to its high prevalence, imposed cost on health systems, and negative impact on patients [8].

Diabetes can be treated, and its complications can be avoided or delayed with diet, physical activity, medications, and regular screening. However, sustained global efforts for improvement must focus on supporting low‐ and middle‐income countries [1]. A united global effort will respond to the increasing burden of diabetes around the world, thereby reducing the risk of diabetes and ensuring that all people diagnosed with the condition have access to equitable, comprehensive, affordable, and quality treatment and care [9].

Similar global endeavors have been mounted to address the epidemic of HIV and significant progress has been made [10]. For instance, the number of children infected with HIV and AID‐related deaths has declined over the years [10, 11]. Access to treatment for HIV has also increased to 28.7 million by 2021 [10]. Given that three in four adults live with diabetes in low and middle‐income countries [4] and it is estimated that 79% of the global diabetic population live in these countries [12]. The burden of the disease is rapidly rising surpassing the existing burden of communicable diseases with unique challenges leaving both adults and children vulnerable to cardiovascular diseases, kidney failure, nerve damage, amputation, vision loss, and deaths [13, 14]. Diabetes have future impact with worrying indications and a major threats to global development as its imposes unacceptably high human, social and economic cost on all income levels in all forms [15].

Bringing coherence to existing complementary efforts to reduce the burden of diabetes for accelerating structural transformation is essential [11]. It is imperative to tackle, prevent, and control the global burden of this condition worldwide, specifically in low‐resource settings. This will bring much positivity in the diabetes world where the risk is reduced and all people with diabetes have access to care [9]. As such, this paper reviews and brings forward novel, collaborative, and integrative strategies to break barriers for better access to diabetes care.

This comprehensive review of the challenges and gaps in access. We developed the following research question: What can be done to secure access to diabetes care for people living with the disease in low‐ and middle‐income countries? We identified novel sustainable and integrative strategies to overcome (break) barriers to better access to diabetes care. In particular, we articulate a clear vision and increase attention on what must be done differently and their global impact on adoption.

## 2 ANALYSIS OF DIABETES CARE ACCESSIBILITY

### 2.1 Overview of pricing and accessibility

According to sources from WHO, access to insulin for four countries in 2019 was 13% [16]. Challenges of unavailability occurred at various levels of the health sector [16]. In the same year, intermediate‐acting insulin in secondary and tertiary hospitals in Peru was 61% and 100%, respectively [16]. In a similar development, a study published in 2019 indicated that the mean availability of insulin in 13 low‐ and middle‐income countries remained between 55% and 88% in a facility where they should be available [17].

**Figure 1:**
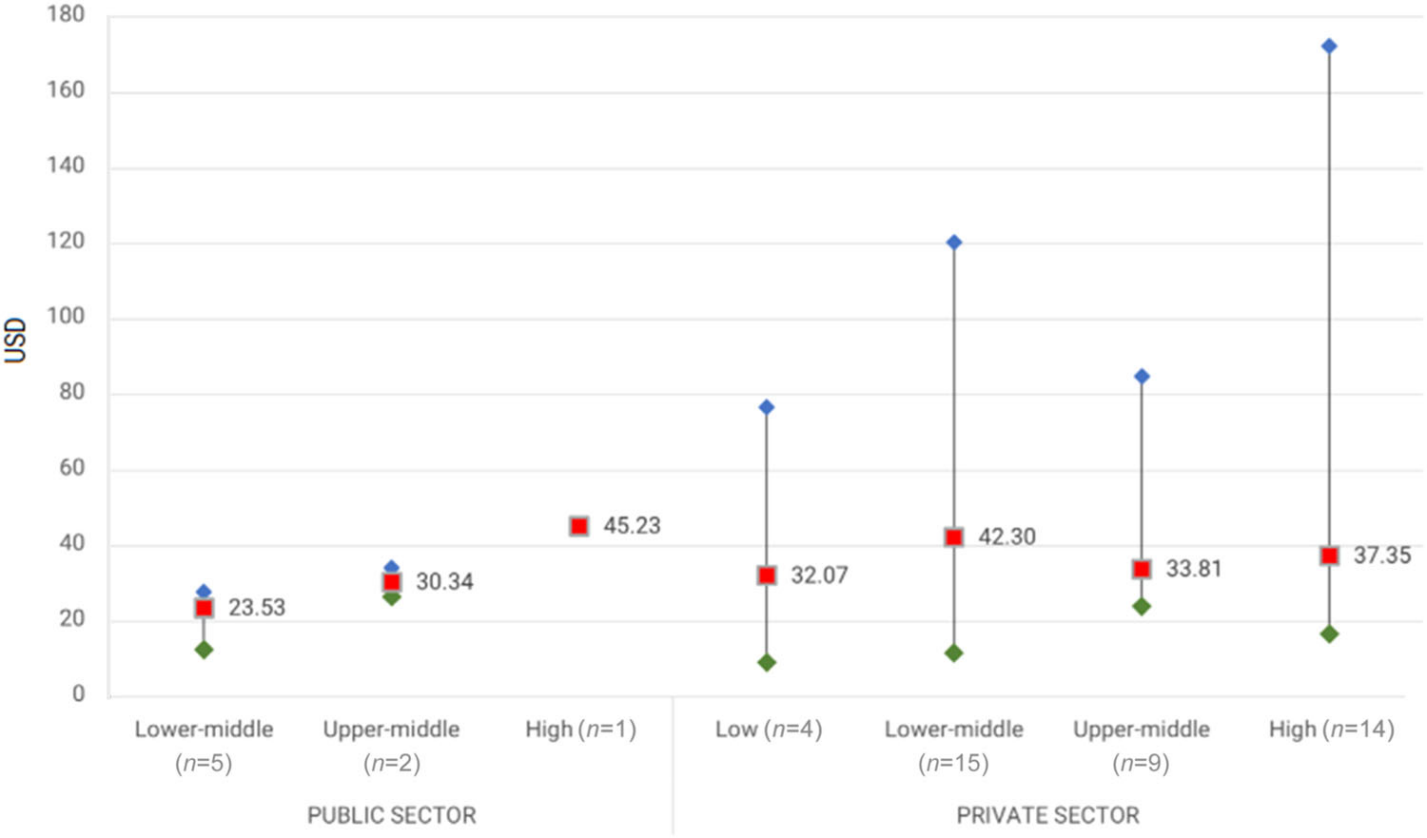
Median and range of insulin prices (glargine 1000 IU) in USD by World Bank Country Income Level. Credit: Health Action International (HAI).

When insulin is available, affordability becomes a challenge for both children and adults [18]. The prices of insulin in resource‐poor settings are among the highest in the world [18] (Figure 1).

Compared with 13.6% of the population in high‐income countries, approximately 35% of patients with diabetes pay higher prices in the private market [18]. In addition, prices vary across low‐resource settings and between countries within the same region [19]. According to a study by Health Action International (Figure 1), a single company has a varied pricing policy from one country to another [19]. For instance, a 15 mg vial of insulin costs 1.5 USD in Iran, whereas the same product may cost over 47 USD in Congo and Namibia, and there is no clear justification for that [19]. In addition, Canadians pay less for the same quantity of insulin than their counterparts in the Palestinian territories [19].

In addition, prices are higher in Africa than in the Eastern Mediterranean and Southeast Asian regions [19]. A recent report issued by the WHO in 2021 summarized the state of global access to insulin as alarming, with low availability, high prices, and few producers dominating the market as the main barriers to universal access [20].

**Figure 2:**
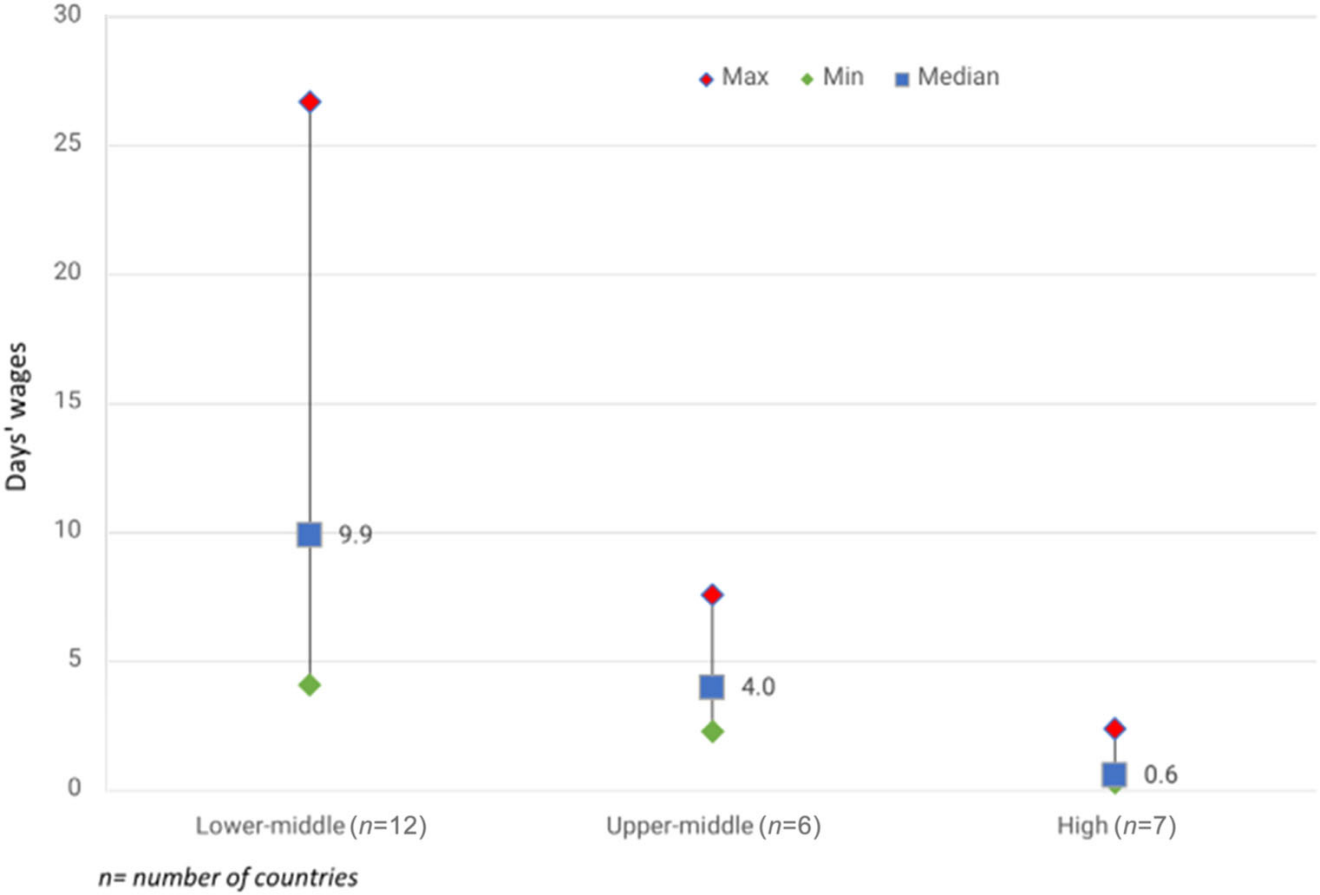
Affordability of insulin (glargine 1000 IU) at the private sector by World Bank Country Income Level. Credit: Health Action International (HAI).

With insulin being the bedrock of diabetes treatment, stakeholders must take steps to close these gaps and expand access to this life‐saving medicine for those who need it before the situation becomes deadly [20]. Diabetes is on the rise in low‐resource countries, and the level of consumption has not met this trend [20] (Figure 2). Therefore, pharmaceutical companies must expand access to insulin in these settings by making it affordable or free [18].

The discovery of insulin 100 years ago was not intended for profit and was sold at an affordable price [20]. Keeping the 100‐year‐old promise involves making it universally accessible [20].

### 2.2 Reasons and factors impacting accessibility

**Figure 3:**
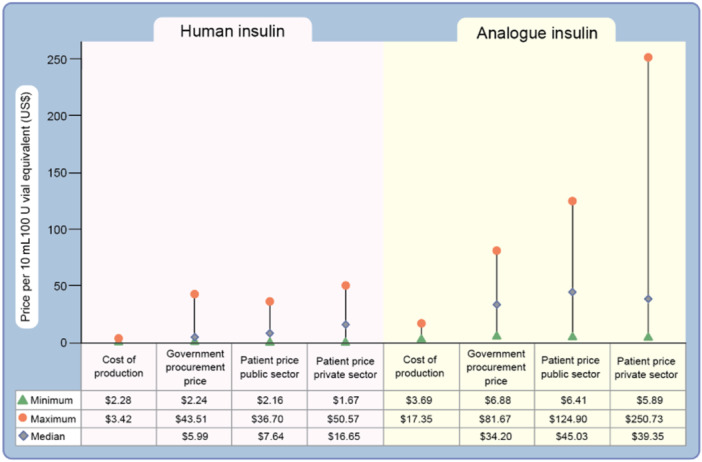
Costs of manufacturing and government procurement, and public and private sector patient prices for human and analog insulin in 43 countries. Credit: Beran et al. (2021) Diabetologia. DOI 10.1007/s00125‐020‐05375‐2 [24]. Copyright license statement: CC.BY allows others to share, copy, and use content, the author does not own the content, and there is an acknowledgment of initial publication/author.

From this article's perspective, these challenges should be tackled from the perspective of fueling the gaps in global access to insulin. A number of known issues keep energizing these challenges. First, the shifting of the global market from human insulin to synthetic (analogous) insulin cannot be ruled out. Human insulin is cheaper to produce than synthetic insulin, which imposes a financial burden on patients in resource‐constrained areas. These analog insulins are 1.5−3 times more expensive than human insulin [20] (Figure 3).

Another study conducted in 2018 estimated that it cost roughly $2−$4 to produce a vial of analog insulin, the one used by most patients [21]. These modern synthetic insulins are patented by large insulin makers, which increases their prices [22]. Consequently, their production is dominated by three multinational companies controlling more than 90% of the insulin market: Eli Lilly, Novo Nordisk, and Sanofil [22]. There is little space for smaller or other companies to compete. These companies also do not sell most of their products in low resource settings because of the limited commercial value of registering and launching them there [18]. These spotlights are exacerbated by poverty, complexities in registration, unstable supply chains and infrastructure to store insulin, and fragile healthcare systems [18].

## 3 INTEGRATED STRATEGIES AND INTERVENTIONS

### 3.1 Marketing

To break the barriers, increase access and attain population affordability. The number, and types of insulin used in poor countries must be expanded. The greater the number of sources a country has, the larger the volume, and the lower the prices tend to be for consumers [22].

According to estimates from the International Diabetes Federation in 2021, out of 537 million people with diabetes worldwide, approximately 80% are from low‐income countries and require insulin therapy [23]. In coupled with this, the commercial value of insulin in these regions varies by type and country, and manufacturer's are unwilling to disclose prices [16]. In 2016, prices from 13 LMICs ranged from $USD 2−25 for human insulin and $USD 22−106 for analog insulin (10 mL vial of 100 IU) compared with data from UK Drug Tariff prices, where LMICs were paying more for analog insulin [16, 17]. In another report made in 2019, median government procurement prices were $5 and $33 for human and analog insulin, respectively, and patients had to pay $34 in public hospitals and $44 in private pharmacies and hospitals for analog insulin [16, 17, 24] (Figure 3).

Although the three main pharmaceutical companies have made significant efforts to offer substantial discounts and free distribution in low‐ and middle‐income nations, such as the 2010 Novo Nordisk Base of the Pyramid Initiative in four African countries. The impact of such programs remains limited, and reductions do not always reach patients [18].

The supply of various products means that there will be more competitive pressure to reduce prices [18]. Drug companies will take steps to focus on the affordable analog insulin as their priority. Market domination by established brands can be minimized [18]. Diabetics can access the best type in terms of the onset of action, peak, duration, concentration, and route [18]. Patients who prefer analog insulin designed to mimic the body's natural release of insulin and carry less risk of hypoglycemia will have access to it [18]. Treatment options available in high‐income countries can also be made possible in poor countries with this intervention. The situation where some countries only have certain types of insulin rather than the array of those in developed nations can be lessened [18]. Much could be solved with this approach to address the affordability challenges of diabetes [18].

### 3.2 Home‐grown production

Manufacturing insulin locally and encouraging competition from smaller local companies are essential. This will ensure a constant supply chain and avoid shortages. Local mass production will ease complex regulatory requirements, authorization, availability, and sales [25]. Subsequently, importation duties, unnecessary taxes, and other add‐up costs for insulin distribution can be avoided [18].

Countries such as China, India, and Russia have increased local manufacturer's and made them active in the market [18]. In China, domestic producers such as Dongbro Pharmaceuticals, Chongqing, Fujin Biology Medical Company, and Gan & Lee pharmaceuticals are major players, and their products are approved by the Chinese Ministry of Human Resources and Social Security on the Insurance Drug List [26]. Through this initiative, a significant number of local manufacturer's have won the bid to supply insulin products to public healthcare institutions across China [27]. Other companies, such as Hefei Tianhui Biotechnology, have succeeded in phase 3 oral insulin clinical trials, submitted a marketing authorization application, and entered a partnership with its US counterpart for commercialization in China [28]. All these developments have contributed to China being currently among the world's largest biosimilar producers.

In India, the number of pharmaceutical industries producing insulin is rapidly growing, as reported by the top 25 biosimilar drug manufacturer's [29]. In Russia, Geropharm produces a local brand called Rinsulin using gene engineering technology that meets domestic and foreign pharmacopeias [30].

However, these efforts alone are not sufficient to achieve universal access to insulin. There must be a commitment to the above set of actions with an approach that promotes alignment with diverse groups, stakeholders, and partners focusing on the global insulin ecosystem.

### 3.3 Solution of policy making

Governments of low‐ and middle‐income countries should collaborate with partners to integrate more accessible efforts into health systems along with the above affordability strategies.

Collaboration is relevant because insulin makers have extensive experience in product delivery and can provide directions, technical know‐how, and resource models for efficient distribution networks, supply, and expansion of cold‐chain capacity [31]. Collaboration and good governance positively affect access by minimizing suboptimal approaches such as high pharmaceutical pricing, weak procurement and supply chain, and insufficient funding to cover demands in these settings [20]. Government‐imposed duties and taxes on imported insulin must be reduced. Lower tariffs lead to lower prices [32]. Priority should be set for health budgets and financial allocation made to ensure affordable insulin to patients in the public sector [33]. Low‐ and middle‐income countries could join pool procurement mechanisms with themselves to negotiate for lower prices instead of procuring them individually at varying prices, leading to high costs [31].

### 3.4 Clinical practice

Adequate forecasting and an efficient supply chain also provide some grounds for hope. Regular survey reports or research on diabetes and the state of cold chain infrastructure are important. The healthcare system will always be aware of the actual demands of insulin, stock‐outs, and levels of product quality needed to treat people living with diabetes [34].

Infrastructure to store insulin, supply of glucose monitoring devices, and syringes to improve use and access will be enhanced. Much could be solved with a better clinical information system [34]. Without these strategies, efforts can easily be touted with any explicit solution, and there will be conflicting priorities.

### 3.5 Health education

Diabetes remains one of the largest global health concerns, burdening public and socioeconomic development [35, 36, 37, 38, 39]. Strategies that address this current demand better than those previously implemented must be considered as we attempt to solve the issues of access to insulin. The creation and enactment of innovative and workable reactions to this burden is necessary. Thus, we must shift our focus from treatment to prevention to have a long‐lasting impact.

Improvement of population health by making it easier for people to live healthy lifestyles, preventing the early onset of the disease, and providing better alternative treatment options address the larger societal problem. Interventions that support patients to become self‐aware of the disease and promote a change in behavior, including increasing health education, will be required.

Clinicians and stakeholders must team up with patients to identify attitudes and behaviors that are harmful or potentially causing the condition and incorporate them into their daily lives [40, 41].

Eating healthy (balanced, fiber rich, carbohydrate controlled, whole grain, limited red meat, and non‐processed), keeping weight in check and staying physically active should be emphasized to the public to prevent pre‐diabetes. Habits such as smoking, alcohol consumption, and saturated fat consumption should be avoided [40, 41].

There must be interactive workshops on the above lifestyle interventions with low literacy patients and the remainder via electronic notifications to phones to provide hands‐on experience [42]. Incentives for patients who are consistent with healthy behaviors and screening programs help optimize the obligation to prevent diabetes.

These awareness campaigns should target communities to increase their understanding of the special needs of people with diabetes. Such an approach will bring about positive change in person behavior and dispel the myths surrounding diabetes [43]. The community will be aware of the different types of preventive measures and how they can be used in different settings for individuals [43].

For patients with diabetes to have access to care, we need more people to be aware that diabetes is real [44]. People with the condition have difficulty in accessing medications, and knowledge in accessing them may not be there [44]. They require assistance from healthcare providers who, in most cases, are not well‐educated and are far from what is required for people living with diabetes [44].

Health education programs can be tailored to the population by diabetes support groups and religious leaders. Diabetes support groups equip patients with knowledge, and action taken from such knowledge produces results and can be part of the integral approach to diabetes management [45].

It further helps vulnerable people and the healthcare system by preventing emergency care and saving money on healthcare costs. Out‐of‐pocket expenditures spent on the procurement of essential diabetes drugs, such as insulin, can be channeled and centered on ultimate beneficiaries, such as poor families, to enable access to insulin free of charge. Shortages and inequalities due to the high demand for insulin by the population could be minimized to provide relief from the cascade of misery and suffering due to lack of access.

Religious leaders also play a critical role. These leaders are important in developing and implementing health interventions [38, 46, 47]. They are community gatekeepers who have access to community members and can deliver information to their congregation [38, 39, 40, 41, 42, 43, 44, 45, 46]. In this regard, religious leaders can be trained to talk about diabetes care, dietary education, and the prevention of gestational diabetes to their congregation. This emphasizes the role of religion as a potential tool and adaptive resource in the problem‐solving process.

### 3.6 Multisectoral approach

Given that the global burden of diabetes has increased significantly since 1990 across different regions and countries, one party alone cannot increase access to care or manage the disease [39].

Work across various sectors, including government agencies, nongovernmental organizations, relevant stakeholders, and other groups, is required to address this practical issue.

A multisectoral approach ensures that resources are efficiently used, efforts complement each other, and are not duplicated [39]. The recognition of diabetes as a cross‐cutting disease where everyone is involved rather than leaving it to health authorities ought to be expressed and advocated by policymakers. People living with diabetes are an obligation of everyone. A cross‐industry approach reduces health disparities by integrating high‐quality care with culturally responsive care [44].

To realize this, the following are some recommendations. We should build a sustainable partnership both within and outside the health sector to address the social and medical factors influencing diabetic care. The diabetes care network must not only be controlled by health experts, but also be expanded to other stakeholders whose engagement is required.

The healthcare system must be redesigned to improve the delivery of diabetes care in a variety of settings (rural and urban); then, key findings and lessons gathered from previous strategies need to be disseminated across these sectors to promote informed decisions. Given the political nature of health, political devotion will support this initiative [45]. Resource‐constrained countries will be better off identifying and addressing the issues of diabetes care by working collaboratively across sectors, contributing to sustainable developmental goals [48]. The same approach has been applied to address many other global health challenges to achieve health equity [49]. Thus, we can postulate the success of achieving global accessibility of therapeutics for diabetes.

## 4 CONCLUSION

Gaps prevail in accessing diabetes care and all forms of insulin. Increasing access is a complex and ongoing challenge in low‐resource settings. Efforts must be continued to improve access to diabetes care.

**Figure 4:**
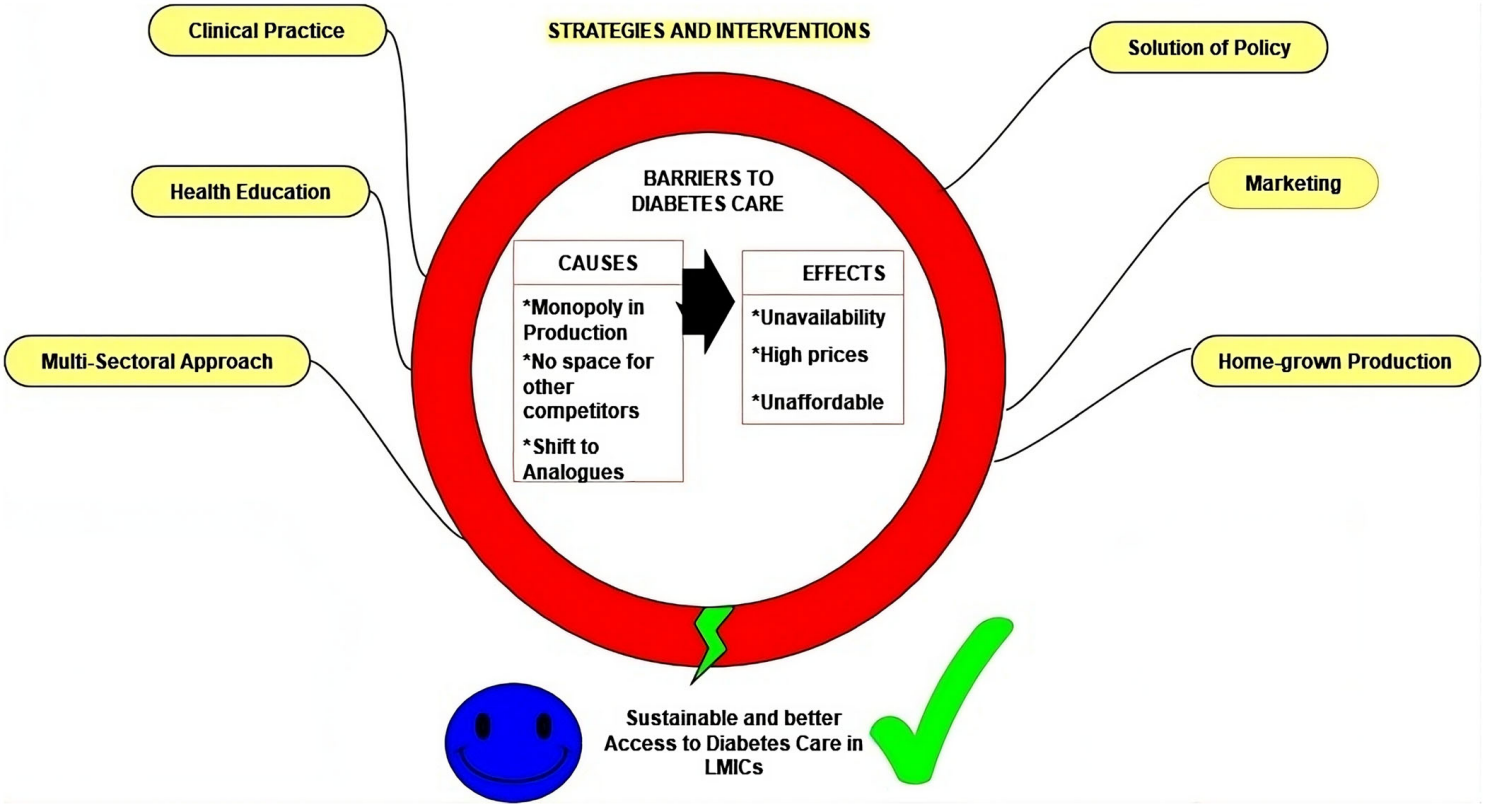
Summary of barriers, impacting factors, and strategetic intervenntions for breaking them.

In this review, we address the question of what can be done to secure access in resource‐constrained settings. We summarized some key insights involving commercial, social, and philanthropic measures to facilitate affordable access to the underserved population (Figure 4). This entails making high‐quality insulin affordable or free of charge and strengthening the healthcare system with innovative strategies to overcome multiple barriers.

## CONFLICT OF INTEREST STATEMENT

The author declares no conflict of interest.

## ETHICS STATEMENT

Not applicable.

## INFORMED CONSENT

Not applicable.

## Data Availability

Data sharing is not applicable to this article as no data sets were generated or analyzed during the current study.
